# Comparison of three health-related quality of life instruments in relation to visual acuity: EQ-5D, 15D, and EUROHIS-QOL8

**DOI:** 10.1007/s11136-022-03293-x

**Published:** 2022-11-17

**Authors:** Petri K. M. Purola, Seppo V. P. Koskinen, Hannu M. T. Uusitalo

**Affiliations:** 1 Department of Ophthalmology, Faculty of Medicine and Health Technology, Tampere University, Arvo Building, 33014 Tampere, Finland; 2 Finnish Register of Visual Impairment, Finnish Federation of the Visually Impaired, Marjaniementie 74, 00930 Helsinki, Finland; 3 Information Services Department of Public Health and Welfare, Finnish Institute for Health and Welfare, Mannerheimintie 166, 00270 Helsinki, Finland; 4 Tays Eye Center, Tampere University Hospital, Biokatu 14, 33520 Tampere, Finland

**Keywords:** Bilateral vision, Epidemiology, Generic measure, Patient-reported outcomes, Population survey, Vision loss

## Abstract

**Purpose:**

To compare three health-related quality of life (HRQoL) instruments in detecting the effect of distance visual acuity (VA) on generic HRQoL in an adult population.

**Methods:**

We used cross-sectional, population-based data from a nationwide health survey conducted in Finland in 2011–2012. It included three self-reported HRQoL instruments, EuroQol-5 Dimension (EQ-5D), 15D, and EUROHIS-QOL8, and a health examination in which habitual distance VA was measured binocularly. We assessed 3764 survey participants aged 30 years and older with information available on these parameters. The comparability and sensitivity of the instruments were evaluated using Pearson correlation coefficients and multivariable linear regression in different VA groups.

**Results:**

EQ-5D and 15D index scores showed strong positive correlation (0.65–0.74) with each other regardless of distance VA, whereas EUROHIS-QOL8 index score showed moderate-to-strong correlation (0.46–0.79) with EQ-5D and 15D. All three instruments showed a negative trend with deteriorating VA, although EQ-5D and 15D showed better sensitivity than EUROHIS-QOL8. When adjusted for age, gender, and co-morbidities, adequate vision (VA 0.63–0.8), weak vision (VA 0.32–0.5), and impaired vision or worse (VA ≤ 0.25) were independently associated with declined EQ-5D and 15D, whereas declined EUROHIS-QOL8 was associated only with adequate and weak vision.

**Conclusion:**

All three instruments can be viable tools in evaluating the relation between vision and HRQoL. While 15D is preferred due to its wide coverage of dimensions, EQ-5D can be an equal alternative, as it has less respondent burden. The feasibility of EUROHIS-QOL8 on detecting differences between lower VA levels may require further evidence.

**Supplementary Information:**

The online version contains supplementary material available at 10.1007/s11136-022-03293-x.

## Introduction

In the recent decades, the use of quality of life (QoL) evaluation from the patients’ perspective has increased in the form of patient-reported outcomes [1, 2]. One such outcome is generic health-related quality of life (HRQoL), which aims to capture the aspects of QoL that can be influenced by health and health care [3]. These include topics related to physical, mental, emotional, and social functioning. Most HRQoL instruments allow these dimensions to be converted into a single generic index score that enables the comparison of HRQoL between different treatments, diseases, and conditions.

Visual impairment is known to have a detrimental effect on QoL, as it is associated with increased difficulties in daily functioning and well-being [4–6]. In fact, even a mild vision loss has been associated with limited functioning and declined QoL [7, 8]. Despite the importance of this subject, there is a paucity of population-based studies on the relation between visual acuity (VA) and generic HRQoL. While many vision-specific QoL instruments have been utilized to assess the relation of vision and eye conditions to QoL, the obtained results are not generalizable to non-eye-related diseases and other factors [9–14]. In addition, the sensitivity of generic HRQoL instruments has been shown inadequate on visual factors in various clinical settings [15, 16].

However, a few population-based studies have shown the potential of generic HRQoL in evaluating vision [7, 17–19], with two HRQoL instruments in particular: EuroQol-5 Dimension [20] and 15D [21]. Therefore, our aim was to provide a comprehensive comparison of commonly used generic HRQoL instruments in relation to distance VA using nationwide, population-based data. We included both EuroQol-5 Dimension and 15D, as well as EUROHIS-QOL8 [22], which to our knowledge has not been previously used in the evaluation of vision at population level.

## Materials and methods

### Health 2011 survey

This study is based on a nationally representative sample of Finnish adults from the Health 2011 Survey conducted by the Finnish Institute for Health and Welfare in 2011–2012 [23]. It is a follow-up study to the Health 2000 Survey conducted in 2000–2001 [24], which included a two-stage stratified cluster sample drawn from the nationwide population register in Finland (*n* = 9922). All participants of the baseline study who were living in Finland in 2011 and had not refused to be invited to further studies were invited to the Health 2011 Survey (*n* = 8135). The study sample included a total of 8006 participants aged 30 years and older. The unweighted participation rates in the baseline and follow-up were 93% and 73%, respectively. The sample weights were calibrated by post-stratification, defined by age, sex, region, and native language to account for non-response and missing data. The details of the survey methods have previously been published [25].

The aim of both surveys was to provide up-to-date information on health, functional capacity, and welfare. Several methods were implemented in both surveys, including self-reported questionnaires, interviews, and a comprehensive health examination. The Health 2011 Survey included three self-reported HRQoL questionnaires: EuroQol-5 Dimension, 15D, and EUROHIS-QOL8. The data on co-morbidities were collected in face-to-face interviews. Distance VA was measured in the health examination.

### Health-related quality of life instruments

The EuroQol-5 Dimension (EQ-5D-3L, later referred as EQ-5D) is a commonly used generic HRQoL instrument [20] that contains one question for each of the five dimensions: mobility, self-care, usual activities, pain/discomfort, and anxiety/depression. Each question contains three answer options on a scale of one (no difficulties) to three (extreme difficulties). In the Health 2011 Survey, only the descriptive system of the instrument was used with the visual analog scale excluded. A single index score is obtained by weighting the obtained scores with population-based preference weights based on an application of the multi-attribute utility theory. The EQ-5D was weighted using the UK time trade-off weights, as it showed very strong correlation (r ≥ 0.9) with Finnish preference weights, and it improves comparability with other populations, particularly European [26]. The EQ-5D index score weighted with UK time trade-off weights has a scale between − 0.59 and 1, with 0 representing HRQoL equal to being dead and 1 representing the best possible HRQoL [27].

The 15D [21] includes 15 questions tapping 15 dimensions: mobility, vision, hearing, breathing, sleeping, eating, speech, excretion, usual activities, mental function, discomfort/symptoms, depression, distress, vitality, and sexual activity. Each dimension/question contains five answer options on a scale of 1 (no difficulties) to 5 (extreme difficulties) that were converted into 15D index scores using Finnish preference weights with a scale of 0 (representing HRQoL equal to being dead) to 1 (representing the best possible HRQoL).

The EUROHIS-QOL8 questionnaire [22] is a derivation of the World Health Organization Quality of Life Instrument-Abbreviated Version (WHOQOL-BREF) [28, 29] that evaluates eight items: overall QoL, general health, energy, daily life activities, self-esteem, social relationships, economic capacity, and habitat. Each item includes an individualized five-point scale with a higher score indicating a better condition. The overall index score is expressed as the mean of the individual scores, ranging from one to five with a higher score indicating a better QoL.

To evaluate the correlation among subscales of the instruments, we combined the subscales that measured parallel dimensions: usual activities (EQ-5D, 15D; daily life activities in EUROHIS-QOL8), vitality (15D; energy in EUROHIS-QOL8), mobility (EQ-5D, 15D), pain/discomfort (EQ-5D; discomfort/symptoms in 15D; general health in EUROHIS-QOL8), and anxiety/depression (EQ-5D; depression and distress in 15D). The subscale scores of EQ-5D and 15D were inverted to standardize the scales with EUROHIS-QOL8 and index scores, i.e., higher score indicates better condition.

### Distance visual acuity

The distance VA was measured in the health examination by an educated study nurse binocularly at 4 m with current visual correction. Illumination was set to ≥ 350 lx on the modified logMAR letter chart [30]. All measurements were standardized. All VA values are presented as Snellen decimal equivalents. Low VA values outside the modified logMAR letter chart that could not be determined were reported as 0.01. Based on the previous studies [7, 18], we classified VA values into following groups: VA ≥ 1.0 (good vision), VA 0.63–0.8 (adequate vision), VA 0.32–0.5 (weak vision), and VA ≤ 0.25 (impaired vision or worse). We found the binocular evaluation of VA important as the relation of vision and HRQoL was investigated.

### Co-morbidities

The survey included an interview with questions on multiple co-morbidities, including eye diseases and other common diseases and disorders. To adjust for potential confounders when investigating the relation of distance VA and HRQoL, we included co-morbidities that had been used in previous studies [7, 18] utilizing the same dataset: glaucoma, unoperated cataract, retinal degeneration, hypertension, diabetes, Parkinson’s disease, unspecified cancer, heart diseases (myocardial infarction, angina pectoris, heart failure, arrhythmias, and “other heart disorders”), pulmonary diseases (asthma, chronic obstructive pulmonary disease, chronic bronchitis, and “other pulmonary disease”), vascular diseases (stroke and varicose veins in lower limbs), musculoskeletal conditions (rheumatoid arthritis, osteoarthrosis, fractures, and osteoporosis), and psychiatric disorders (psychotic disorders, depression, anxiety, psychoactive substance abuse, and “other psychiatric disease”). Participants were assumed to not have a co-morbidity on a missing value if they had answered to at least one of the co-morbidity questions. Unoperated cataract patients were compared to persons without cataract or with operated cataract, as cataract surgery is known to improve VA, as well as QoL [31].

### Statistical analyses

All analyses were performed using R software (v. 4.1.2, R Core Team, R Foundation for Statistical Computing, Austria). The sampling design in the survey was accounted for using Survey package 3.37 for R [32] and weighting scheme calculated by the Finnish Institute for Health and Welfare. Persons with missing data in QoL subscale analyses (*n* = 56 in 15D, *n* = 104 in EUROHIS-QOL8) and multivariable regression analyses (*n* = 41) were excluded. Correlations were calculated with Pearson correlation coefficient using function svycor in jtools package 2.1.4 [33], which is an increment to the Survey package in accounting for the sampling design. Standard errors for the coefficients were calculated using function wtd.cor in weights package 1.0.4 [34] with a bootstrap procedure using the number of participants in the analysis. Correlations were evaluated using Evans’ classification [35]. Because the HRQoL data were left-skewed, Kruskal–Wallis test was used to compare HRQoL index scores and subscale scores, adjusted with Dunn–Bonferroni correction. The impact of age, gender, distance VA, and co-morbidities on HRQoL was estimated through a multivariable linear regression. The Tobit model was also created for EQ-5D to account for its skewed distribution and ceiling effect using censReg package 0.5-36 [36, 37], but the results did not differ significantly from the current model. Multicollinearity was measured through variance inflation factors using car package 2.1-5 for R [38, 39]. All predictors resulted in values below 2, therefore showing no indication of collinearity. For all analyses, a two-tailed *p* value of < 0.05 was considered as statistically significant.

## Results

Of the eligible 8006 invited participants aged 30 years and older, 3764 (47%) had information available for all three HRQoL index scores and distance VA, and therefore were included in the analyses. More details of the study population are shown in Table 1.

**Table 1:** Summary of the Health 2011 study population aged 30 years and older

	*n*	Mean age (SD)	% Women
Eligible sample	8006	55 (16)	53
HRQoL and distance VA known	3764	56 (14)	57
Distance VA ≥ 1.0	3151	53 (12)	57
Distance VA 0.63–0.8	467	66 (13)	55
Distance VA 0.32–0.5	121	71 (12)	57
Distance VA ≤ 0.25	25	74 (15)	68

*SD* standard deviation, *HRQoL* health-related quality of life, *VA* visual acuity

The correlation between EQ-5D, 15D, and EUROHIS-QOL8 among different VA groups is shown in Table 2. EQ-5D and 15D showed strong positive correlation with each other in all VA groups. EUROHIS-QOL8 showed moderate positive correlation with both instruments, with strong correlation among those with impaired vision or worse.

**Table 2:** Correlation between EQ-5D, 15D, and EUROHIS-QOL8 index scores in different distance visual acuity (VA) groups with 95% confidence intervals

	EQ-5D vs. 15D	EQ-5D vs. EUROHIS-QOL8	15D vs. EUROHIS-QOL8
All	0.68*** (0.66–0.71)	0.51** (0.48–0.54)	0.59** (0.57–0.62)
VA ≥ 1.0	0.66*** (0.64–0.69)	0.50** (0.47–0.53)	0.59** (0.56–0.62)
VA 0.63–0.8	0.65*** (0.59–0.71)	0.46** (0.37–0.54)	0.57** (0.49–0.65)
VA 0.32–0.5	0.67*** (0.55–0.78)	0.46** (0.32–0.60)	0.52** (0.35–0.69)
VA ≤ 0.25	0.74*** (0.49–0.99)	0.79*** (0.65–0.94)	0.74*** (0.58–0.90)

Correlation was calculated using Pearson correlation coefficient

*Denotes weak correlation based on Evan’s classification

**Denotes moderate correlation based on Evan’s classification

***Denotes strong or very strong correlation based on Evan’s classification

The correlation based on parallel subscales between the instruments is shown in Table 3. Between EQ-5D and 15D, usual activities, mobility, and anxiety/depression showed strong positive correlation, whereas pain/discomfort showed mostly moderate correlation. EUROHIS-QOL8 showed mostly moderate positive correlation between the two instruments based on usual activities and vitality, with pain/discomfort the weakest. As was observed for the index scores, EUROHIS-QOL8 subscales showed strong correlation with both other instruments among those with impaired vision or worse.

**Table 3:** Correlation between EQ-5D, 15D, and EUROHIS-QOL8 subscales in different distance visual acuity (VA) groups

	EQ-5D vs. 15D					EQ-5D vs. EUROHIS-QOL8		15D vs. EUROHIS-QOL8		
	Usual activities	Mobility	Pain/discomfort	Anxiety/depression^a^	Anxiety/depression^b^	Usual activities	Pain/discomfort	Usual activities	Vitality	Pain/discomfort
All	0.68***	0.62***	0.58**	0.62***	0.56**	0.48**	0.44**	0.49**	0.60***	0.45**
VA ≥ 1.0	0.65***	0.61***	0.59**	0.59**	0.56**	0.46**	0.44**	0.47**	0.60***	0.45**
VA 0.63–0.8	0.67***	0.59**	0.51**	0.70***	0.58**	0.46**	0.42**	0.48**	0.54**	0.42**
VA 0.32–0.5	0.71***	0.61***	0.60***	0.61***	0.47**	0.43**	0.29*	0.49**	0.48**	0.28*
VA ≤ 0.25	0.65***	0.42**	0.56**	0.87***	0.73***	0.62***	0.57**	0.78***	0.91***	0.69***

Correlation was calculated using Pearson correlation coefficient

^a^Depression dimension in 15D

^b^Distress dimension in 15D

*Denotes weak correlation based on Evan’s classification

**Denotes moderate correlation based on Evan’s classification

***Denotes strong or very strong correlation based on Evan’s classification

**Fig. 1:**
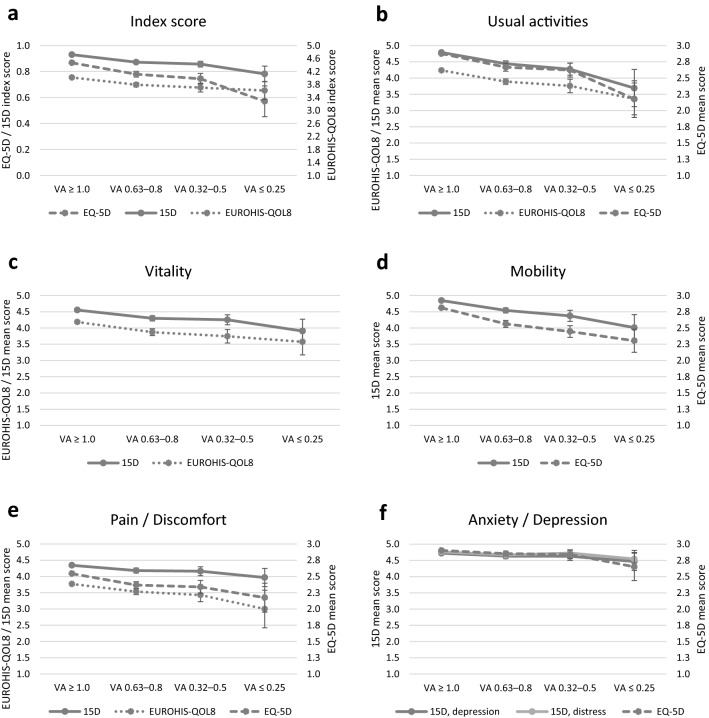
Relation between health-related quality of life and distance visual acuity (VA) based on EQ-5D, 15D and EUROHIS-QOL8 index score and subscale means with 95% confidence intervals. Scales of all three instruments have been standardized, i.e., higher score indicates better overall quality of life or condition. Statistical significances are included in Online Resource 1

The relation of the index score and subscale score means between the three instruments among different VA groups is visualized in Fig. 1 (for numerical presentation see table in Online Resource 1). Deteriorating VA showed a negative trend in index and subscale scores of all three HRQoL instruments. These trends were evaluated using correlations and statistical significance. Correlations between each instrument and VA groups were 0.22 [95% confidence interval (CI) 0.18–0.27] for EQ-5D, 0.29 (95% CI 0.25–0.33) for 15D, and 0.15 (95% CI 0.11–0.18) for EUROHIS-QOL8; therefore, all three instruments showed weak correlation with VA groups. When the statistical significance of the scores was evaluated (see table in Online Resource 2), all three instruments showed significant difference between the good vision group and the three declined vision groups (adequate, weak, and impaired or worse) according to the index scores; however, all three instruments showed mostly low sensitivity in detecting differences between the three declined vision groups. When the subscale scores were evaluated, the association between declining HRQoL and declining vision was most significant in usual activities and mobility, whereas the association was weakest in pain/discomfort and anxiety/depression.

To investigate the association between the three HRQoL instruments and distance VA in relation to co-morbidities and other factors, we utilized a multivariable regression analysis. The regression was performed for each HRQoL instrument separately, using three different distance VA cutoff values: VA ≤ 0.25, ≤ 0.5, and ≤ 0.8. The results for each cutoff group are reported in Table 4. All cutoff values were associated with significantly declined EQ-5D and 15D index scores, with 15D showing the best fit. On the other hand, EUROHIS-QOL8 showed significant decline only with cutoff values ≤ 0.5, and ≤ 0.8. EUROHIS-QOL8 also showed the weakest fit. Overall, the impact on HRQoL was more severe the lower the cutoff value was.

**Table 4:** Multivariable linear regression analysis examining the impact of distance visual acuity (VA) at different cutoff values, age, gender, and co-morbidities on EQ-5D, 15D, and EUROHIS-QOL8 index scores

	Change in EQ-5D		Change in 15D		Change in EUROHIS-QOL8	
	B coefficient	Beta coefficient	B coefficient	Beta coefficient	B coefficient	Beta coefficient
Constant	1.002***		0.996***		4.122***	
Distance VA ≥ 1.0	Ref.		Ref.		Ref.	
Distance VA ≤ 0.8	− 0.035**	− 0.064**	− 0.028***	− 0.111***	− 0.150***	− 0.082***
Distance VA ≤ 0.5	− 0.052*	− 0.051*	− 0.032**	− 0.067**	− 0.229**	− 0.066**
Distance VA ≤ 0.25	− 0.160**	− 0.071**	− 0.088**	− 0.084**	− 0.191	− 0.025
Age	− 0.001***	− 0.100***	− 0.001***	− 0.109***	0.002*	0.050*
Male gender	0.012*	0.033*	0.003	0.018	− 0.052**	− 0.043**
Glaucoma	− 0.023	− 0.022	− 0.016	− 0.033	0.039	0.011
Cataract, unoperated	0.017	0.020	− 0.012	− 0.032	0.023	0.008
Retinal degeneration	− 0.005	− 0.005	− 0.007	− 0.015	0.022	0.007
Heart disease	− 0.051***	− 0.101***	− 0.029***	− 0.124***	− 0.141***	− 0.083***
Pulmonary disease	− 0.036**	− 0.071**	− 0.028***	− 0.117***	− 0.109**	− 0.063**
Vascular disease	− 0.008	− 0.016	− 0.005	− 0.020	− 0.078**	− 0.045**
Musculoskeletal condition	− 0.069***	− 0.193***	− 0.018***	− 0.109***	− 0.102***	− 0.084***
Hypertension	− 0.037***	− 0.096***	− 0.017***	− 0.094***	− 0.139***	− 0.105***
Diabetes	− 0.041**	− 0.063**	− 0.025**	− 0.082**	− 0.144**	− 0.065**
Psychiatric disorder	− 0.124***	− 0.215***	− 0.073***	− 0.273***	− 0.559***	− 0.286***
Parkinson’s disease	− 0.123	− 0.035	− 0.052**	− 0.032**	− 0.156	− 0.013
Cancer	0.002	0.003	− 0.008	− 0.025	− 0.042	− 0.018
R^2^	0.209***		0.286***		0.155***	
Adjusted R^2^	0.205***		0.283***		0.151***	
Null deviance	126		27		1424	
Residual deviance	100		19		1203	

The unstandardized B coefficients show the magnitude of the impact on health-related quality of life while the standardized Beta coefficients allow the comparison of the explanatory variables with each other. The analysis was based on participants with information available for all predictors (*n* = 3723)

*Denotes statistical significance with *p* < 0.05

**Denotes statistical significance with *p* < 0.01

***Denotes statistical significance with *p* < 0.0001

## Discussion

To our knowledge, this is the first population-based study to investigate the comparability and sensitivity of EQ-5D, 15D, and EUROHIS-QOL8 on detecting the impact of distance VA on generic HRQoL. In summary, EQ-5D and 15D showed strong positive correlation with each other in all distance VA levels, whereas EUROHIS-QOL8 showed mostly moderate correlation with both EQ-5D and 15D. Of the observed subscales, usual activities showed the strongest correlation between all three instruments. All three instruments presented a negative trend with declining VA, although EQ-5D and 15D were more sensitive in detecting differences between lower VA levels, even after adjusting for age, gender, and co-morbidities.

These results compare well with the previous studies on Health 2000 and 2011 data. Taipale et al. reported that distance VA showed linear trend with both EQ-5D and 15D [7]. We reported that visual impairment had stronger impact on EQ-5D and 15D than the awareness of vision-threatening eye diseases [18, 40]. In the current study, we have observed a similar trend using EUROHIS-QOL8.

While population-based studies on this subject are scarce, at least two studies utilizing population representative data have shown similar association between EQ-5D and visual impairment [17, 19]. Both Park et al. and Wu et al. reported that EQ-5D was able to identify significant differences between groups with different severity of visual impairment. To our knowledge, there are no population-based studies conducted in other countries than Finland that have utilized 15D or EUROHIS-QOL8 on evaluating vision, although there are studies that have utilized other HRQoL instruments, such as Medical Outcomes Study 12-Item Short-Form Health Survey [41] and 36-item short form health survey [6, 42].

The correlation between EQ-5D and 15D has been shown to vary between different diseases and conditions [43–45]. 15D has usually been observed as the more sensitive instrument, likely due to its larger number of items and levels [46]. EQ-5D has been reported to be less sensitive in better health states, possibly due to its known ceiling effect [47]. Nevertheless, in this study, EQ-5D and 15D showed a strong correlation with each other, particularly among those with impaired vision or worse. On the other hand, EUROHIS-QOL8 showed mostly moderate correlation with EQ-5D and 15D. Interestingly, all three instruments showed the best correlation among those with impaired vision or worse. A possible explanation could be that this group is more homogenous in relation to HRQoL than those with mild vision loss or normal vision.

In the previous clinical-based studies, the association between generic HRQoL instruments and visual factors has been inconsistent [15, 16, 48]. However, in this population-based study, all three instruments showed a negative trend with declining VA levels. The greatest difference was usually found between good vision (VA ≥ 1.0) and adequate vision (VA 0.63–0.8), whereas the differences were somewhat more equalized between lower VA levels. Those with adequate vision may feel significant discomfort, particularly if the loss of visual ability was acute, whereas those with moderate and severe vision loss may have already adapted to their current state and no longer feel similar discomfort.

Of the evaluated parallel subscales, usual activities and mobility showed the strongest association with declining vision. This was expected, as vision is known to play a significant role in these dimensions [5, 49]. Furthermore, three out of the five dimensions of EQ-5D—mobility, self-care, and usual activities—have shown strong association with vision loss in the previous studies [7, 18]. This likely explains the sensitive capability of EQ-5D on VA comparable to that of 15D despite not including a similar vision dimension.

There has been discussion on whether a vision “bolt-on” may increase the responsiveness in EQ-5D with some preliminary evidence supporting its use in cost–utility analyses [50, 51]. However, the additional vision dimension may lead to different valuations of the five EQ-5D dimensions; hence, without appropriate high-quality valuation of each bolt-on dimension, the inclusion of bolt-ons could potentially reduce the comparability between studies that have included different bolt-on dimensions and those without any bolt-ons [50]. The inclusion of vision bolt-on may also affect the relationship between visual impairment and other co-morbidities. Therefore, based on our study, EQ-5D is sufficiently sensitive to detect the impact of vision on HRQoL in a population-based sample even without validated and standardized bolt-on vision-related dimensions.

After adjusting for age, gender, and co-morbidities in a multivariable regression model, EQ-5D and 15D showed independent association with severe and mild forms of vision loss, whereas EUROHIS-QOL8 was independently associated only with mild forms due to its high variance in the severe form. Therefore, EQ-5D and 15D appear to be more sensitive to visual impairment than EUROHIS-QOL8. However, it should be noted that the number of participants with severe vision loss (VA ≤ 0.25) was low (*n* = 25); hence, the sensitivity of EUROHIS-QOL8 should be tested in a larger sample to confirm this. Our model included vision-threatening eye diseases—glaucoma, unoperated cataract, and retinal degeneration—which can be interpreted as the cause for the outcome (vision loss). However, no significant differences were observed in the results or the fitness of the model when all three eye diseases were excluded; therefore, we saw appropriate to include the eye diseases in the analyses. This supports our previous findings, in which the visual impairment was stronger determinant of declined HRQoL than the awareness of an eye disease [18].

All in all, in this population-based study, we show that EQ-5D, 15D, and EUROHIS-QOL8 can be feasible tools in evaluating the impact of vision on generic HRQoL. This is particularly noteworthy for EQ-5D, as it shows almost equal sensitivity to 15D, but is more compact and widely used. We also show that a specific vision dimension may not be required for a HRQoL instrument to be able to detect different levels of VA, as vision can impact HRQoL through other dimensions, usually related to activities of daily living, self-care, and mobility. EUROHIS-QOL8 also appears a viable alternative, although more population-based studies are required to confirm its sensitivity on lower distance VA levels.

The greatest strength of this study is the nationwide population sample that represents the Finnish adult population. The Health 2011 Survey and the baseline survey addressed public health issues more comprehensively than national health surveys do on average, allowing the inclusion of multiple HRQoL instruments and large number of co-morbidities in the analyses. The participation rate in the present study can be considered good, and the loss to follow-up was compensated by applying a calibrated weighting scheme [25]. All analyses were conducted on the largest possible number of participants, with population sampling weights compensating for the limitations of complete case only analyses. Furthermore, the study data did not consist of specific patient groups collected from health-care units allowing for better generalization of the results.

There are also potential limitations in our study. While our findings are based on a dataset from ten years ago, the strengths of the survey mentioned previously should compensate this. Based on the low participation among participants younger than 30 years and the rarity of vision problems in this age group, we decided to leave it out from our study. Visual field and contrast sensitivity were not measured in the health examination, and therefore were not included in the determination of visual impairment. However, their impact on EQ-5D, 15D, and EUROHIS-QOL8 has remained uncertain in clinical settings, and there is even less evidence at population level [15, 16, 48]. Co-morbidities were self-reported, and therefore subject to bias. The selection of the parallel subscales of the three instruments was based solely on the similarity of the dimensions, and therefore the parallel subscales may not be entirely comparable due to differences in the presentation of the questions and answer scales. This was particularly noticeable in the pain/discomfort dimension, because EUROHIS-QOL8 included a question about general health rather than the more specific pain and discomfort dimensions in EQ-5D and 15D. However, the correlations between other parallel subscales were from modest to strong, indicating good comparability. The regression models were associated with low R-squared scores. Previous studies based on the same dataset have also reported low R-squared scores when evaluating HRQoL instruments [7, 18]. This is likely due to the complicated and subjective nature of these instruments. Because the study population was Finnish, the results may not be directly applicable to other countries and ethnicities, although our use of UK time-trade-off weights for EQ-5D is likely to improve comparability, as it is commonly used in other European countries [26]. In addition, the UK tariffs for EQ-5D showed strong correlation with Finnish tariffs, which should indicate good comparability with 15D with the Finnish preference weights. However, no specific weights were applied to EUROHIS-QOL8, which may partially explain its weaker comparability to both EQ-5D and 15D.

In conclusion, EQ-5D, 15D, and EUROHIS-QOL8 are comparable with each other on different vision levels, showing moderate-to-strong correlation. EQ-5D and 15D showed high sensitivity to vision, whereas EUROHIS-QOL8 showed more variance, particularly among those with severely impaired vision. Therefore, all three instruments can be viable tools in evaluating the relation between vision and HRQoL. The 15D is preferred due to its comprehensive and most sensitive nature, but EQ-5D can be an equal alternative, as it has less respondent burden. Furthermore, a bolt-on vision dimension may not be necessary in an HRQoL instrument, if it includes other dimensions impacted by vision, such as usual activities and mobility. In future studies, more population-based samples could be used to confirm whether the results are reproducible in other populations, particularly the sensitivity of EUROHIS-QOL8 on low vision levels.

## Supplementary Information

Below is the link to the electronic supplementary material.

Supplementary file1 (PDF 142 kb)

Supplementary file2 (PDF 151 kb)

## Data Availability

Full study protocol, contact details, publications, and the process for collaborating and data requests can be found on the website (thl.fi/health2011).
