# Supplementary material for: Comparison of three health-related quality of life instruments in relation to visual acuity: EQ-5D, 15D, and EUROHIS-QOL8
Source: Qual Life Res. 2022 Nov 17;32(2):543–52. doi: 10.1007/s11136-022-03293-x (PMC9911483; doi:10.1007/s11136-022-03293-x)
Supplement: Supplementary file 1 — Supplementary file1 (PDF 142 kb) [file 11136_2022_3293_MOESM1_ESM.pdf]

Comparison of three health-related quality of life instruments in relation to visual acuity – EQ-5D, 15D, and EUROHIS-QOL8

Quality of Life Research

Petri K M Purola, Seppo V P Koskinen, and Hannu M T Uusitalo

Corresponding author: Petri K M Purola

Department of Ophthalmology, Faculty of Medicine and Health Technology, Tampere University, Tampere, Finland

Finnish Register of Visual Impairment, Finnish Federation of the Visually Impaired, Helsinki, Finland

Email: petri.purola@tuni.fi

Online Resource 1 Index score and subscale score means (and standard errors) shown in Fig. 1

	Index score			Usual activities			Vitality		Mobility		Pain / Discomfort			Anxiety / Depression		
	EQ-5D	15D	EUROHIS-QOL8	EQ-5D	15D	EUROHIS-QOL8	15D	EUROHIS-QOL8	EQ-5D	15D	EQ-5D	15D	EUROHIS-QOL8	EQ-5D	15D, depression	15D, distress
VA ≥ 1.0	0.87 (0.00)	0.93 (0.00)	4.0 (0.0)	2.9 (0.0)	4.8 (0.0)	4.2 (0.0)	4.6 (0.0)	4.2 (0.0)	2.8 (0.0)	4.8 (0.0)	2.5 (0.0)	4.3 (0.0)	3.8 (0.0)	2.9 (0.0)	4.7 (0.0)	4.8 (0.0)
VA 0.63–0.8	0.78 (0.01)	0.87 (0.01)	3.8 (0.0)	2.7 (0.0)	4.4 (0.1)	3.9 (0.0)	4.3 (0.0)	3.9 (0.1)	2.6 (0.0)	4.5 (0.0)	2.4 (0.0)	4.2 (0.0)	3.5 (0.1)	2.9 (0.0)	4.6 (0.0)	4.7 (0.0)
VA 0.32–0.5	0.74 (0.02)	0.86 (0.01)	3.7 (0.1)	2.6 (0.1)	4.3 (0.1)	3.8 (0.1)	4.3 (0.1)	3.7 (0.1)	2.4 (0.1)	4.4 (0.1)	2.3 (0.1)	4.2 (0.1)	3.4 (0.1)	2.8 (0.0)	4.6 (0.1)	4.7 (0.1)
VA ≤ 0.25	0.57 (0.06)	0.78 (0.03)	3.6 (0.1)	2.2 (0.1)	3.7 (0.3)	3.4 (0.3)	3.9 (0.2)	3.6 (0.2)	2.3 (0.1)	4.0 (0.2)	2.2 (0.1)	4.0 (0.1)	3.0 (0.3)	2.7 (0.1)	4.5 (0.1)	4.5 (0.1)

Scales of all three instruments have been standardized, i.e., higher score indicates better overall quality of life or condition. *VA* visual acuity.
