# Supplementary material for: Comparison of three health-related quality of life instruments in relation to visual acuity: EQ-5D, 15D, and EUROHIS-QOL8
Source: Qual Life Res. 2022 Nov 17;32(2):543–52. doi: 10.1007/s11136-022-03293-x (PMC9911483; doi:10.1007/s11136-022-03293-x)
Supplement: Supplementary file 2 — Supplementary file2 (PDF 151 kb) [file 11136_2022_3293_MOESM2_ESM.pdf]

Comparison of three health-related quality of life instruments in relation to visual acuity – EQ-5D, 15D, and EUROHIS-QOL8

Quality of Life Research

Petri K M Purola, Seppo V P Koskinen, and Hannu M T Uusitalo

Corresponding author: Petri K M Purola

Department of Ophthalmology, Faculty of Medicine and Health Technology, Tampere University, Tampere, Finland

Finnish Register of Visual Impairment, Finnish Federation of the Visually Impaired, Helsinki, Finland

Email: petri.purola@tuni.fi

Online Resource 2 Statistical significance for index scores and subscale scores shown in Fig. 1

	Index score, p			Usual activities, p			Vitality, p		Mobility, p		Pain / Discomfort, p			Anxiety / Depression, p		
	EQ-5D	15D	EUROHIS-QOL8	EQ-5D	15D	EUROHIS-QOL8	15D	EUROHIS-QOL8	EQ-5D	15D	EQ-5D	15D	EUROHIS-QOL8	EQ-5D	15D, depression	15D, distress
VA 0.63–0.8 vs. VA ≥ 1.0	**<0.0001**	**<0.0001**	**<0.0001**	**<0.0001**	**<0.0001**	**<0.0001**	**<0.0001**	**<0.0001**	**<0.0001**	**<0.0001**	**<0.0001**	**0.0003**	**<0.0001**	0.1594	0.1984	**0.0022**
VA 0.32–0.5 vs. VA ≥ 1.0	**<0.0001**	**<0.0001**	**<0.0001**	**<0.0001**	**<0.0001**	**<0.0001**	**<0.0001**	**<0.0001**	**<0.0001**	**<0.0001**	**0.0004**	0.0531	**0.0013**	0.1613	0.3862	1.0000
VA ≤ 0.25 vs. VA ≥ 1.0	**<0.0001**	**<0.0001**	**0.0460**	**<0.0001**	**<0.0001**	**0.0023**	**0.0030**	0.0627	**<0.0001**	**<0.0001**	**0.0162**	0.1244	0.1264	**0.0058**	0.2585	0.0923
VA 0.32–0.5 vs. VA 0.63–0.8	0.6787	0.4131	0.5430	1.0000	0.3201	1.0000	1.0000	0.3760	**0.0460**	0.0754	1.0000	1.0000	1.0000	1.0000	1.0000	1.0000
VA ≤ 0.25 vs. VA 0.63–0.8	**0.0136**	**0.0406**	1.0000	**<0.0001**	**0.0342**	0.8735	0.6290	1.0000	**0.0181**	**0.0223**	0.8798	1.0000	1.0000	**0.0423**	0.8603	0.7930
VA ≤ 0.25 vs. VA 0.32–0.5	0.2059	0.5512	1.0000	**<0.0001**	0.5271	1.0000	1.0000	1.0000	0.7520	0.7008	1.0000	1.0000	1.0000	0.2225	1.0000	0.4035

P-values were calculated using Kruskal–Wallis test adjusted with Dunn–Bonferroni correction. Bolded values denote statistical significance with $p < 0.05$. *VA* visual acuity.
